# Implications of stimulus-induced, rhythmic, periodic, or ictal discharges (SIRPIDs) in hospitalized patients

**DOI:** 10.3389/fneur.2022.1062330

**Published:** 2023-01-23

**Authors:** Paola Martinez, Irfan Sheikh, M. Brandon Westover, Sahar F. Zafar

**Affiliations:** Department of Neurology, Massachusetts General Hospital, Boston, MA, United States

**Keywords:** seizures, SIRPIDs, critically ill, electroencephalography, stimulus induced, GPDs, LPDs

## Abstract

**Background:**

Stimulus-induced electroencephalographic (EEG) patterns are commonly seen in acutely ill patients undergoing continuous EEG monitoring. Despite ongoing investigations, the pathophysiology, therapeutic and prognostic significance of stimulus-induced rhythmic, periodic or ictal discharges (SIRPIDs) and how it applies to specific pathologies remain unclear. We aimed to investigate the clinical implications of SIRPIDs in hospitalized patients.

**Methods:**

This is a retrospective single-center study of hospitalized patients from May 2016 to August 2017. We included patients above the age of 18 years who underwent >16 h of EEG monitoring during a single admission. We excluded patients with cardiac arrest and anoxic brain injury. Demographic data were obtained as well as admission GCS, and discharge modified Rankin Score (mRS). EEGs were reviewed for background activity in addition to epileptiform, periodic, and rhythmic patterns. The presence or absence of SIRPIDs was recorded. Our outcome was discharge mRS defined as good outcome, mRS 0–4, and poor outcome mRS, 5–6.

**Results:**

A total of 351 patients were included in the final analysis. The median age was 63 years and 175 (50%) were women. SIRPIDs were identified in 82 patients (23.4%). Patients with SIRPIDs had a median initial GCS of 12 (IQR, 6–15) and a length of stay of 12 days (IQR, 6–15). They were more likely to have absent posterior dominant rhythm, decreased reactivity, and more likely to have spontaneous periodic and rhythmic patterns and higher frequency of burst suppression. After adjusting for baseline clinical variables, underlying disease type and severity, and EEG background features, the presence of SIRPIDs was also associated with poor outcomes classified as MRS 5 or 6 (OR 4.75 [2.74–8.24] *p* ≤ 0.0001).

**Conclusion:**

In our cohort of hospitalized patients excluding anoxic brain injury, SIRPIDs were identified in 23.4% and were seen most commonly in patients with primary systemic illness. We found SIRPIDs were independently associated with poor neurologic outcomes. Several studies are indicated to validate these findings and determine the risks vs. benefits of anti-seizure treatment.

## 1. Introduction

Stimulus-induced electroencephalographic (EEG) patterns are commonly seen in acutely ill patients undergoing continuous EEG monitoring (1–3). The American Clinical Neurophysiology Society (ACNS) has defined these patterns as stimulus-induced rhythmic delta activity, periodic discharges, spike, and wave discharges, ictal-interictal continuum patterns, brief ictal rhythmic discharges, and seizures (4). Collectively these patterns are referred to as stimulus-induced rhythmic, periodic, or ictal-appearing discharges (SIRPIDs) (4). SIRPIDs have been reported with an incidence of 10–34% (1–3, 5, 6), and can be seen in patients with acute brain injuries (e.g., trauma, stroke, and infections), anoxic brain injury, epilepsy, neurodegenerative diseases and toxic-metabolic disturbances (6, 7). Despite ongoing research, the pathophysiology, therapeutic and prognostic significance of SIRPIDs continues to be uncertain and it is unclear how it applies to specific pathologies. In a large cohort, SIRPIDs were not associated with an increased risk of seizures (8). However, small cohorts examining the association of SIRPIDs with mortality and functional outcomes have shown conflicting results and have included patients with anoxic brain injury/post-cardiac arrest pathology (1–3, 5, 6). Given anoxic brain injury/post-cardiac arrest patients represent a unique pathophysiology and entity, often with a worse prognosis, we aimed to focus our study on patients excluding anoxic brain injury as an etiology for decreased consciousness. The goal of this study was to describe the relationship of SIRPIDs with neurologic outcomes in a cohort of acutely ill patients undergoing EEG monitoring.

## 2. Methods

This is a retrospective cohort study of patients admitted to a single center between May 2016 and April 2017. The study was approved by the Institutional Review Board. Informed consent was not required. The results are reported in accordance with the Strengthening the Reporting of Observational Studies in Epidemiology (STROBE) guidelines for reporting observational studies (9). The data that support the findings of this study are available from the senior author upon reasonable request. We included patients who were above the age of 18 years and who underwent >16 h of EEG monitoring where the duration of consecutive artifacts is < 30% of the total length. We excluded patients with cardiac arrest.

### 2.1. Patient demographics

Data were extracted including age, gender, primary admitting diagnosis, GCS score on admission, history of epilepsy, hospital length of stay, in-hospital mortality, use of anti-seizure medications (ASMs) during hospital stay, and use of anesthetic drugs, discharge location.

### 2.2. EEG

The EEG recordings were obtained using the international 10-20 system. Per institutional protocol, all EEGs were reviewed and reported by two clinical neurophysiologists. All EEG findings were reported using the American Clinical Neurophysiology Society nomenclature (ACNS) (10). The relevant EEG data were subsequently abstracted from the clinical EEG reports. Reports were reviewed for the best background activity (alpha, beta, theta, delta, or burst suppression) and unilateral vs. focal slowing, presence of sleep architecture, sharp waves, generalized periodic discharges (GPD), lateralized periodic discharges (LPDs), generalized rhythmic delta activity (GRDA), lateralized rhythmic delta activity (LRDA), bilateral independent periodic discharges (BIPD), brief ictal rhythmic discharges (BIRDS), seizures (electrographic and clinical), and SIRPIDs. If SIRPIDs were present, further data were collected to ascertain which pattern type i.e., stimulus-induced (SI) patterns, SI-GPD, SI-LPD, SI-GRDA, SI-LRDA, SI-BIPD, and SI-seizures.

### 2.3. Outcomes

We examined discharge neurologic status as measured by the modified Rankin Scale (mRS); 0—no symptoms, 1—no significant disability, 2—slight disability, 3—moderate disability, 4—moderately severe disability, 5—severe disability, and 6—death (11). We defined poor neurologic outcome as mRS of 5 to 6. mRS was abstracted from a physician and physical and occupational therapy clinical examinations by reviewers who were blinded to the EEG findings as previously described (12).

### 2.4. Statistical analysis

For descriptive statistics, we calculated mean, median, and interquartile ranges. Fisher's exact test was used for the comparison of dichotomized and categorical variables, and the Mann-Whitney U-test was used for continuous variables. Significance was set at 0.05, and two-sided *p*-values were reported. We performed a multivariate logistic regression analysis to assess the relationship between SIRPIDs and discharge outcomes. We adjusted for baseline variables including age, sex, and underlying diagnosis. We adjusted for the Glasgow Coma Scale (GCS) as a marker for disease severity. We also adjusted for the presence of spontaneous epileptiform abnormalities (LPDs, GPDs, LRDA, sporadic discharges that were not stimulus-induced), the presence of burst suppression (more than 50% of the record consisting of attenuation or suppression with alternating bursts) (4), and poor EEG background (absent PDR, or absent sleep architecture or absent reactivity) (4). Odds ratios and 95% confidence intervals (OR [95% CI]) were calculated to quantify the association of SIRPIDs with outcomes. The goodness-of-fit for logistic regression models was assessed using the Hosmer–Lemeshow test.

## 3. Results

**Figure 1:**
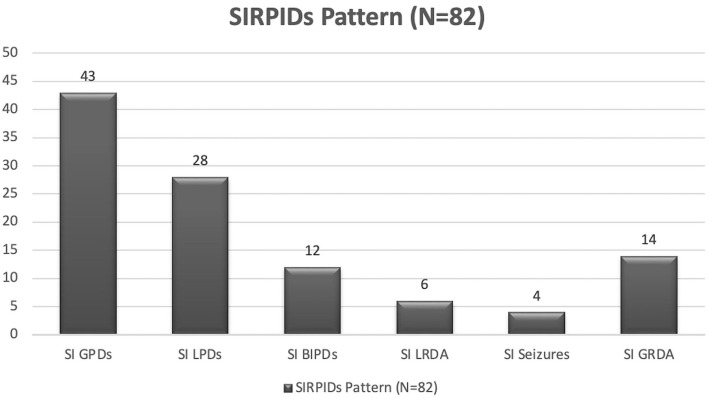
SIRPIDs EEG patterns.

A total of 351 patients were included in the final analysis. Baseline characteristics are presented in Table 1. The median age of the cohort was 63 (IQR, 52–74 years,), and 175 (49.8%) were women, of which 82 (23.4%) patients had SIRPIDs. Patients with SIRPIDs were older (median age 70 years (Q1–Q3, 60–79) vs. 63 years (Q1–Q3, 52–74) in patients without SIRPIDs). Patients admitted with a primary systemic illness, and those with a history of epilepsy were more likely to have SIRPIDs. Patients with SIRPIDs were more likely to have absent PDR and decreased reactivity on EEG. Patients with SIRPIDs were also more likely to have spontaneous periodic and rhythmic patterns, and a higher frequency of burst suppression compared to patients without SIRPIDs. There was no significant difference in the frequency of clinical seizures between patients with SIRPIDs vs. without. Interestingly, patients with SIRPIDs were more likely to have electrographic status epilepticus. The distribution of stimulus-induced pattern types is shown in Figure 1. GPDs were the most common stimulus-induced pattern.

**Table 1:** Baseline characteristics and outcomes.

	**All patients (*N =* 351)**	**Patients with SIRPIDS**	**Patients without SIRPIDS**	***p*-value**
		**(*****N** =* **82)**	**(*****N** =* **269)**	
**Age (median, Q1–Q3)**	63 (52–74)	70 (60–79)	62 (49–72)	< 0.0001
**Gender, Female (%)**	175 (49.8%)	49 (60%)	126 (47%)	0.044
**History of stroke**	84 (24.7%)	23 (28%)	64 (24%)	0.466
**History of hypertension**	175 (49.8%)	49 (60%)	126 (47%)	0.044
**History of epilepsy**	85 (24.4%)	11 (13%)	74 (28%)	0.0082
**History of brain surgery**	41 (11.7%)	5 (6%)	36 (13%)	0.079
**History of CNS malignancy**	38 (10.8%)	5 (6%)	33 (12%)	0.1544
**History of dementia**	19 (5.4%)	4 (5%)	15 (9%)	1
**Initial GCS (median, Q1–Q3)**	14 (8–15)	12 (6–15)	14 (9–15)	0.0329
**Clinical seizures**	38 (11%)	9 (11%)	29 (11%)	1
Use of ASMs	307 (87%)	74 (90%)	233 (87%)	0.4506
DC on ASMs	221 (63%)	41 (50%)	179 (67%)	0.0089
Length of stay (median, Q1–Q3)	14 (8–25.5)	12 (6–15)	12 (7–20)	< 0.0001
**Primary diagnosis**				
CVA	71 (20.2%)	22 (27%)	49 (18%)	0.115
TBI	42 (11.9%	9 (11%)	33 (12%)	0.8476
NeuroID/Inflam	22 (6.2%)	7 (9%)	15 (6%)	0.301
NeuroOnc	39 (11.1%)	3 (4%)	36 (13%)	0.0147
Other Neuro	42 (11.9%)	9 (11%)	33(8%)	0.848
Primary Systemic	68 (19.3%)	24 (29%)	44 (16%)	0.0159
Seizure/Status	67 (19%)	8 (10%)	59 (22%)	0.0154
**DC mRS**				< 0.0001
0	14 (4%)	1 (1%)	13 (5%)	
1	11 (3%)	0	11 (4%)	
2	12 (3%)	1 (1%)	11 (4%)	
3	39 (11%)	4 (5%)	35 (13%)	
4	112 (32%)	15 (18%)	97 (36%)	
5	102 (29%)	36 (44%)	66 (25%)	
6	61 (17%)	25 (30%)	36 (13%)	
**EEG characteristics**				
Burst suppression on EEG	45 (13%)	24 (29%)	21 (8%)	< 0.0001
PDR on EEG	126 (36%)	12 (15%)	114 (42%)	< 0.0001
Sleep architecture	113 (32%)	15 (18%)	98 (36%)	0.0019
EEG reactivity	170 (48%)	31 (38%)	139 (52%)	0.032
EEG sporadic sharps	237 (67%)	69 (84%)	168 (62%)	0.0002
GPDs	100 (28%)	52 (63%)	48 (18%)	< 0.0001
LPDs	135 (38%)	40 (49%)	95 (35%)	0.0376
GRDA	106 (30%)	33 (40%)	73 (27%)	0.028
LRDA	65 (19%)	23 (28%)	42 (16%)	0.0147
BiPDs	50 (14%)	20 (24%)	30 (11%)	0.006
EEG status	25 (7%)	11 (13%)	14 (5%)	0.0239
Electrographic seizures	66 (19%)	23 (28%)	43 (62%)	0.0229
Electrographic status	25 (7%)	11 (13%)	14 (5%)	0.0239

### 3.1. Outcomes

**Figure 2:**
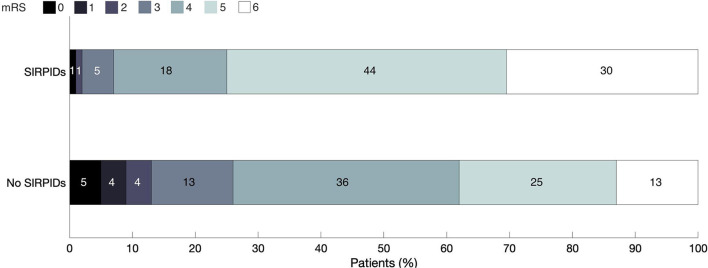
Distribution of discharge modified Rankin Scale scores across the cohort.

The distribution of discharge mRS scores across the cohort is shown in Figure 2. On univariate analysis presence of SIRPIDs was in poor neurologic outcome (OR 4.76 [2.74–8.24] *p* ≤ 0.0001). After adjusting for baseline variables, and other EEG features (presence of epileptiform abnormalities, burst suppression, and poor background), SIRPIDs continued to be significantly associated with poor outcomes defined as mRS of 5–6 (OR 2.41 [1.27–4.60], *p* = 0.007).

### 3.2. Sensitivity/Subgroup analyses

Sensitivity analysis was performed in patients with epileptiform abnormalities such as seizures, periodic discharges, or rhythmic delta activity. In the subgroup of patients with epileptiform abnormalities, SIRPIDs continued to be associated with poor outcomes, even after adjusting for baseline variables (OR 2.94 [160–5.42] *p* = 0.0005). We performed an additional sensitivity analysis including anti-seizure medications (ASMs) in the regression model. After adjusting for ASM use, SIRPIDs continued to be significantly associated with poor outcomes (OR 2.45 [CI 1.29–4.63], *p* = 0.0006).

## 4. Discussion

In our cohort of hospitalized patients, SIRPIDs were seen in 24% of patients and occurred more commonly in patients with primary systemic illness. We found that SIRPIDs were independently associated with poor discharge outcomes (8). In light of our findings, larger studies are indicated to confirm our findings and determine the optimal treatment strategies including anti-seizure medication treatment vs. minimizing frequent stimuli that result in SIRPIDs.

The prevalence of SIRPIDs (23.4%) in our study is comparable to prior literature (1–3, 5, 13). Previously published studies have conflicting findings on the association of SIRPIDs with outcomes. A study of post-cardiac arrest patients found SIRPIDs were associated with poor prognosis if they were seen in conjunction with intermittent or unreactive EEG background activity (14). In another study of post-cardiac arrest patients' absence of reactivity to external stimuli or absence of a posterior dominant rhythm were associated with death or persistent coma at discharge, while SIRPIDs were not significantly associated with outcomes (5). In a larger series of 416 patients, age, anoxic brain injury, and lack of EEG reactivity were independently associated with in-hospital mortality, while SIRPIDs were not (3). A potential explanation for our different findings from prior work is that we excluded patients with cardiac arrest, while all prior studies have either specifically focused on post-cardiac arrest patients or included anoxic brain injury, a disease subgroup with a distinct prognostic profile.

The median GCS of patients with SIRPIDs was 12 (6–10, 12–15) demonstrating SIRPIDs can be seen across a spectrum of disease severities, and not limited to severe brain injury as previously thought (1, 5). SIRPIDs were seen most commonly in patients with primary systemic illnesses, 24/82 (29%), and may be secondary to the underlying metabolic process. We also found that the most common stimulus-induced pattern was generalized periodic discharges (SI-GPDs) which were seen in 43 (52%) of patients with stimulus-induced patterns. Given the majority of our patients with SIRPIDs were those with primary systemic illnesses, it is not unexpected that the most common SI pattern observed in our study was SI-GPDs. GPDs are commonly associated with metabolic derangements (15) and a majority of patients with GPDs have a toxic-metabolic illness or sepsis and may have a coexisting brain injury (16–19). Therefore, another treatment consideration is correcting metabolic derangements, in addition to or as an alternative to anti-seizure treatments.

We found SIRPIDs were more likely to be present if the EEG also showed spontaneous periodic and rhythmic patterns. Periodic and rhythmic patterns have been shown to be associated with increased metabolic stress and secondary brain injury that may worsen outcomes (20–22). The exact mechanism underlying stimulus-induced ictal patterns is not entirely understood, and studies have suggested a component of hyperactivity within the thalamocortical system (23) and an additional hypothesis that relates to the dorsal midbrain anticonvulsant zone (DMAZ) which seems to play a role in brainstem networks related to seizures (24). Further work is needed to understand the underlying mechanisms of SIRPIDs, and to determine whether they exert metabolic stress similar to spontaneous ictal patterns.

Interestingly, we found our patients with SIRPIDs were more likely to have electrographic status. Similar to the association with outcomes there are variable reports on the association of SIRPIDs with seizures, with some studies showing no association between SIRPIDs and seizures (1, 25), while others have found SIRPIDs associated with focal motor and non-convulsive seizures (2, 3, 15, 24). However, these studies had a smaller number of patients with SIRPIDS and did not account for anti-seizure treatment and whether increasing ASMs in response to SIRPIDs may reduce the subsequent risk of electrographic seizures.

There were several limitations of this study including its retrospective nature and being a single-center study. with a small sample size. We did not account for ASM use in our analysis, as it is difficult to disentangle the indication for ASM (clinic seizures vs. spontaneous EEG findings vs. SIRPIDS). While we adjusted for multiple confounders, there may be residual unmeasured confounding.

## 5. Conclusion

In summary, in a cohort of acutely ill patients, the presence of SIRPIDs was significantly associated with poor outcomes defined. The decision to treat continues to be challenging and further prospective studies will be needed to determine if antiseizure medications or minimizing stimuli is the best treatment approach.

## Data availability statement

The raw data supporting the conclusions of this article will be made available by the authors, without undue reservation.

## Author contributions

The first draft of the manuscript was written by PM and IS and all authors commented on, edited, and revised previous and final versions of the manuscript. All authors contributed to the study's conception and design. Material preparation, data collection, and analysis were performed by all authors. All authors read and approved the final manuscript.
